# Manual versus Automated Carotid Artery Plaque Component Segmentation in High and Lower Quality 3.0 Tesla MRI Scans

**DOI:** 10.1371/journal.pone.0164267

**Published:** 2016-12-08

**Authors:** Loek P. Smits, Diederik F. van Wijk, Raphael Duivenvoorden, Dongxiang Xu, Chun Yuan, Erik S. Stroes, Aart J. Nederveen

**Affiliations:** 1 Department of Vascular Medicine, Academic Medical Center, Amsterdam, the Netherlands; 2 Department of Radiology, University of Washington, Seattle, United States of America; 3 Department of Radiology, Academic Medical Center, Amsterdam, the Netherlands; Northwestern University Feinberg School of Medicine, UNITED STATES

## Abstract

**Purpose:**

To study the interscan reproducibility of manual versus automated segmentation of carotid artery plaque components, and the agreement between both methods, in high and lower quality MRI scans.

**Methods:**

24 patients with 30–70% carotid artery stenosis were planned for 3T carotid MRI, followed by a rescan within 1 month. A multicontrast protocol (T1w,T2w, PDw and TOF sequences) was used. After co-registration and delineation of the lumen and outer wall, segmentation of plaque components (lipid-rich necrotic cores (LRNC) and calcifications) was performed both manually and automated. Scan quality was assessed using a visual quality scale.

**Results:**

Agreement for the detection of LRNC (*Cohen’s* kappa (*k)* is 0.04) and calcification (*k* = 0.41) between both manual and automated segmentation methods was poor. In the high-quality scans (visual quality score ≥ 3), the agreement between manual and automated segmentation increased to *k* = *0*.55 and *k* = 0.58 for, respectively, the detection of LRNC and calcification larger than 1 mm^2^. Both manual and automated analysis showed good interscan reproducibility for the quantification of LRNC (intraclass correlation coefficient (ICC) of 0.94 and 0.80 respectively) and calcified plaque area (ICC of 0.95 and 0.77, respectively).

**Conclusion:**

Agreement between manual and automated segmentation of LRNC and calcifications was poor, despite a good interscan reproducibility of both methods. The agreement between both methods increased to moderate in high quality scans. These findings indicate that image quality is a critical determinant of the performance of both manual and automated segmentation of carotid artery plaque components.

## Introduction

Based on randomized controlled clinical trials[1,2], current guidelines recommend surgical treatment (carotid endarterectomy) for symptomatic severe carotid artery stenosis (70%-99%)[3]. Due to the relatively high risk of complications, surgical therapy is mainly beneficial in patients at high risk for recurrent stroke. For patients with a moderate (<70%) symptomatic carotid artery stenosis, guidelines therefore advise medical treatment, consisting of lipid-lowering, antihypertensive and antiplatelet medication[3]. Despite optimal medical treatment, patients with moderate carotid artery stenosis are still at risk for recurrent stroke.

Insights in the individual patient risk for recurrent stroke can aid in the clinical decision for surgical or medical treatment. Besides luminal stenosis grade, measurement of other plaque specific characteristics (i.e. plaque composition, fibrous cap thickness, inflammatory activity[4]) may help in identification of high risk patients. Multicontrast carotid Magnetic Resonance Imaging (MRI) allows non-invasively assessment of plaque composition[5,6]. The identification of certain carotid artery plaque components by MRI (specifically intraplaque hemorrhage (IPH), lipid-rich necrotic core (LRNC) and calcifications), detected by MRI, were able to predict future ischemic stroke in several studies [7–11]. Currently, larger prospective multicenter studies are running to investigate the role of MRI-based plaque characterization in clinical risk-stratification models to predict (recurrent) ipsilateral stroke(PARISK[12]), and to aid in the choice for surgical or medical treatment in symptomatic carotid artery stenosis <70% (ECST-2, ISRCTN# 97744893).

Clinical implementation of carotid MRI for risk stratification in patients with carotid artery stenosis requires accurate, reproducible and high-throughput evaluation of MR-images of arterial wall plaques. The variability in neck size and location of the vessels relative to the skin may however in practice lead to a broad spectrum in image quality. To date, analysis of plaque components is predominantly performed manually [13]. For widespread implementation of carotid plaque component analysis (for example as an outcome parameter in large multicenter studies, or for the clinical decision whether or not to perform carotid endarterectomy), rapid and reliable analysis is essential. Automation of the analysis may aid in meeting these requirements. The findings in recent studies suggesting that fully automated plaque component analysis software (PlaqueView) may be as accurate and reproducible as the aforementioned manual analysis[14,15] are thus encouraging. We, however, hypothesize that image quality is a critical determinant of the accuracy and reproducibility of automated segmentation of plaque components.

In the present paper, we therefore studied the agreement between manual versus automated plaque component segmentation and compared the reproducibility of both methods in patients with moderate (30–70%) carotid artery stenosis, and. In addition, we explored the impact of MR image quality on both the reproducibility of, and the agreement between both methods.

## Methods

This observational single center (Academic Medical Center Amsterdam) study was conducted in concordance with Good Clinical Practice guidelines. The study protocol was approved by the local investigational review board (Medical Ethical Committee–Academic Medical Center Amsterdam) and written informed consent was obtained from all participants. As the current study used patient and MRI data from a previous study, patient selection and most study procedures are described in detail in previous publications [16,17]. In short, patients with a 30–70% carotid artery stenosis on ultrasound were included for a 3T-MRI scan of the carotid artery, followed by a rescan within 1 month. For the MRI scans, a 3T whole-body MRI scan (Intera, Philips Medical Systems, Best, The Netherlands) combined with a 8 channel dedicated bilateral carotid artery coil (Shanghai Chenguang Medical Technologies, Shanghai, China) was used. High resolution (0.25 by 0.25 mm) T1w, T2w, PDw and TOF images were acquired centered around the area with the most profound plaque burden, using ECG-gated unilateral axial sequences (imaging parameters in S1 Table [17]). Overview images of the planning scans were used to plan the repeat scan. Analysis of plaque composition was performed manually and automated for all included patients.

### Image analysis

Before any quantitative analysis was performed, one reader (RD, 3 years of experience with carotid MRI) manually corrected all scan and rescan images for possible Z-axis displacement using T1w, PDw and TOF images in VesselMass. All scans and rescans were checked for co-registration using the carotid bifurcation as a localizer.

For manual analysis, vessel wall dimensions and components (areas with lipid rich necrotic core (LRNC) and calcifications) were analysed by one reader (DFvW, 3 years of experience with carotid MRI) using dedicated software (VesselMass, Leiden) [18]. First, the lumen and outer wall were delineated. For the analysis of plaque components, all four weightings (T1w, T2w, PDw and TOF) were used to identify areas of LRNC and calcifications. Iso-intense to hyperintense areas on T1w and PDw images with varying intensities on T2w and TOF images were considered to correspond with the LRNC. Calcification was defined by a hypo-intense signal on all four weightings[5]. LRNC and calcification dimensions were displayed as mean wall area (MWA) (mm2) per slice.

**Fig 1:**
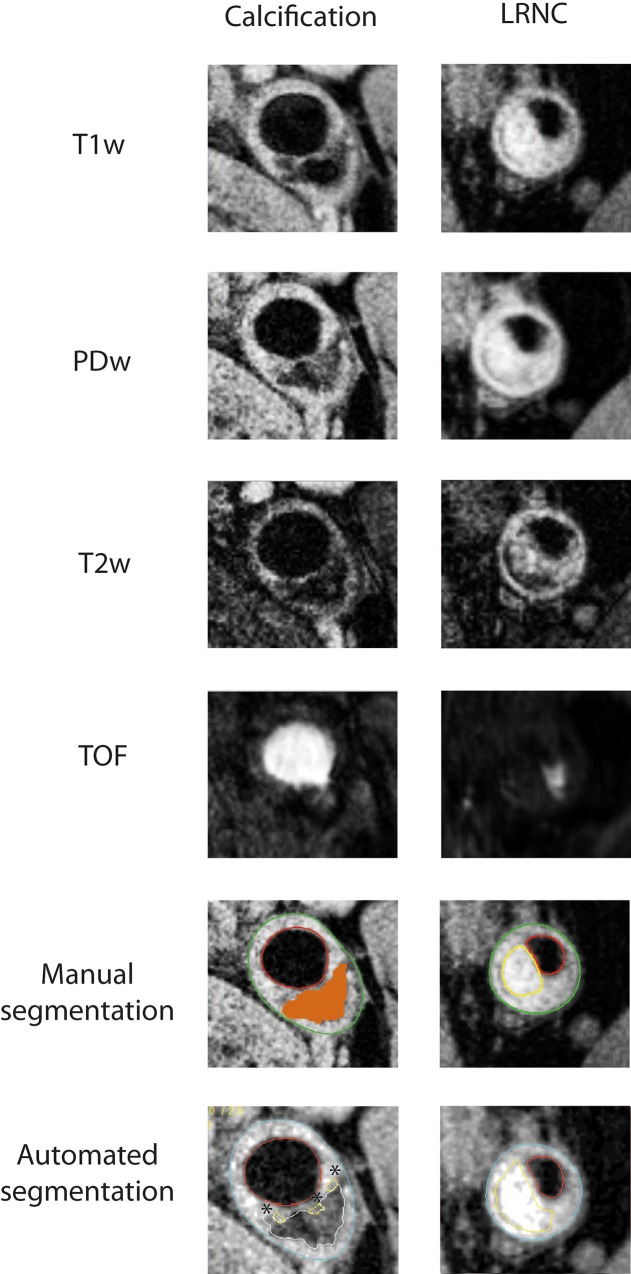
Representative images of manual and automated segmentation of LRNC and calcifications. Representative images of the manual and automated segmentation of a calcified plaque area and a lipid-rich necrotic core (LRNC) using a multicontrast MRI protocol of the carotid artery. Shown are all the individual MRI sequences (T1w,PDw,T2w,TOF), as well as the manual and automated analysis. Lumen contours were delineated in red for both methods, and outer wall contours were delineated in green for manual segmentation, and light blue for automated segmentation. Calcified plaque areas were coloured orange in manual segmentation, and delineated white in automated segmentation. LRNCs were delineated yellow in both manual and automated segmentation. In these examples, both methods agree on the identification of a large calcified plaque area (left example) and large LRNC (right example). Please also note the identification of three small LRNC areas using automated segmentation (*), which are not detected by manual segmentation.

Automatic analysis of vessel wall components was performed by one reader (LPS) using the PlaqueView software (VP diagnostics, Seattle, USA), an automated program for segmentation of vessel wall components using the MEPPS algorithm [15]. Four weightings (T1w, T2w, PDw and TOF) were used for plaque components analysis. First, lumen and outer wall contours were delineated automatically, with the possibility of the reader to manually correct, followed by co-registration of the different contrast weightings. The delineation of areas with LRNC or calcification was then performed fully automated using the MEPPS algorithm[19]). Correction of automated segmented areas of LRNC or calcification was not allowed. LRNC and calcification dimensions were displayed as MWA per slice (mm2). Fig 1 shows representative images of manual and automated segmentation of a LRNC area and a calcified plaque.

Manual analysis of plaque components was performed using both open (during analysis, the reader was able to see both the scan and rescan) and closed segmentation (during analysis, the reader was blinded for the baseline or follow-up scan). Closed segmentation was used to calculate the interscan reproducibility of manual segmentation, whereas the manual open segmentation was used to study the agreement between manual and automated analysis.

### Scan quality

To assess scan quality as a parameter influencing reproducibility, all images were scored according to a visual quality score from 1 (poor) to 4 (excellent), based on the ability to delineate the outer wall and lumen (1, arterial wall margins unidentifiable; 2, arterial wall is visible, but lumen and outer boundaries are indistinct; 3, arterial wall structures are identifiable, but lumen and outer boundaries are not totally clear; 4, arterial wall and lumen are well defined). The mean visual quality score from the scan and rescan was calculated. A mean score of ≥ 3 was defined as a high quality scan, a score < 3 was defined as a low quality scan. The between reader intraclass correlation coefficient for the visual quality score in the present study was 0.77 (0.46–0.90, reflecting a good reproducibility.

### Statistical analysis

Continuous variables are expressed as mean ± SD. Quantitative agreement between the successive MRI measurement of LRNC and calcification plaque area was assessed using intra-class correlation coefficients (ICC). Only MRI scans from subjects containing the specific plaque component in the scan and/or rescan in automated and/or manual analysis were included in this analysis. An ICC of <0.40 indicated poor, one between 0.40 and 0.75 indicated fair to good, and one of >0.75 indicated excellent reproducibility. The agreement between manual and automated detection of a LRNC-containing and calcified plaque was assessed with using Cohen’s kappa (k, with 0.00–0.20 = slight agreement, 0.21–0.40 = fair agreement, 0.41–0.6 = moderate agreement, 0.61–0.80 = substantial agreement, 0.81–1.00 = near-perfect agreement[20]). This analysis was repeated for large plaque components (only LRNC or calcifications with a MWA of > 1mm^2^ included). Both the reproducibility of each method, as well as the agreement between both methods, were stratified for scan quality (high quality scans versus low quality scans). All statistical analyses were performed using PASW statistics 18.0 for Windows (SPSS Inc., Chicago, IL, USA).

## Results

### Participants

Fifty-one individuals with one or more atherosclerotic events were screened for the presence of atherosclerotic carotid artery disease using ultrasound. Thirty-one individuals with a 30 to 70% stenosis of the carotid artery were included in the study protocol. Of those, seven subjects were excluded due to absence of a rescan (n = 4), or because automated plaque component analysis could not be performed (n = 3), resulting in a total of twenty-four subjects for our analysis (48% female, mean age 68 years).

Using manual segmentation, LRNC were detected in carotid artery plaques of in 6/24 (25%) of the included subjects. A substantially higher number was found using automated segmentation: 23/24 (96%) of plaques contained a LRNC. Also after exclusion of small areas of LRNC (< 1 mm^2^ plaque area per slice), automated segmentation still identified higher numbers of LRNC containing plaques compared to the manual analysis (14/24 in automated analysis versus 5/24 in manual analysis). Calcified plaques were found in 19/24 subjects using manual segmentation, compared to 22/24 using automated segmentation. After exclusion of small areas of calcification (MWA <1mm^2^ per slice), 17/24 plaques contained calcifications in the manual analysis, and 12/24 in automated analysis. In neither manual nor automated analysis, IPH containing plaques were found.

**Fig 2:**
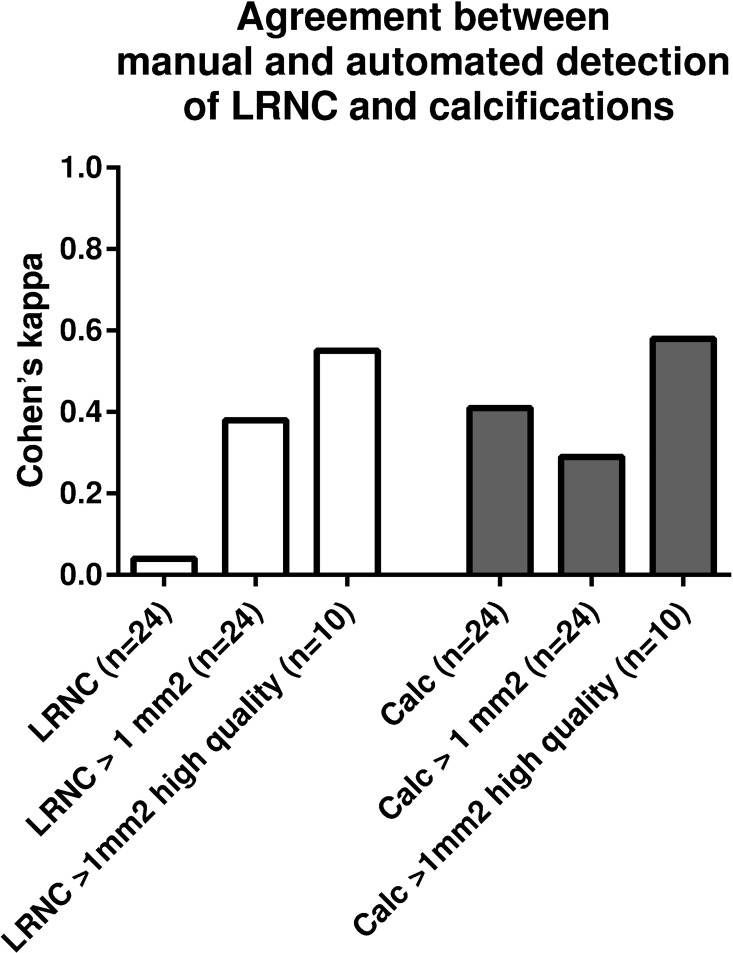
Agreement between manual and automated detection of plaque components. Agreement between the detection of LRNC- and calcification- containing plaques by manual and automated analysis. Cohen’s kappa values for agreement between manual and automated analysis are shown for all plaque components in all scans; plaque components > 1 mm^2^ in all scans; and plaque components > 1 mm^2^ in high quality scans only.

Subsequently, we explored the agreement (using Cohen’s *k*) between the manual and automated detection of plaques containing LRNC and/or calcifications (Fig 2). We found a poor agreement for the detection of LRNC (*k* = 0.04) and a fair agreement for the detection of a calcified plaque (*k* = 0.41). When plaque components < 1 mm^2^ were excluded from analysis, the *kappa* increased slightly for LRNC detection (to *k* = 0.38), but decreased for calcified plaque detection (to *k* = 0.29). When only high quality scans (10 scans with a visual quality score of 3 or more) were included, agreement between manual and automated detection of large plaque components increased to *k =* 0.55 for LRNC and *k =* 0.58 for calcified plaques, reflecting moderate agreement.

### Reproducibility of manual and automated plaque component analysis

Both the manual and automated analysis showed good interscan reproducibility for the *quantification* of LRNC plaque area (ICC of 0.80 and 0.94 respectively) and calcified plaque area (ICC of 0.77 and 0.95 respectively) (Table 1). Overall, interscan reproducibility was higher for the automated segmentation. In the lower quality scans (visual quality score < 3) the interscan reproducibility for the quantification of LRNC and calcification remained good in automated segmentation (0.92 for LRNC, 0.95 for calcification), whereas this was markedly reduced in the manual segmentation (0.60 for LRNC and 0.69 for calcification).

**Table 1:** Interscan reproducibility of quantification of plaque components using manual and automated segmentation.

	Interscan ICCall scans (n = 24)	Interscan ICC high quality scans (n = 10)	Interscan ICC lower quality scans (n = 14
**LRNC**			
*Automated segmentation*	0.94 (0.87–0.98)	0.98 (0.94–1.00)	0.92 (0.75–0.98)
*Manual segmentation*	0.80 (0.52–0.91)	0.90 (0.61–0.98)	0.60 (0.00–0.88)
**Calcifications**			
*Automated segmentation*	0.95 (0.89–0.98)	0.98 (0.90–0.99)	0.90 (0.70–0.97)
*Manual segmentation*	0.77 (0.48–0.90)	0.82 (0.27–0.96)	0.69 (0.02–0.90)

ICC = intraclass correlation coefficient; LRNC = lipid-rich necrotic core

### Post-hoc analysis

To evaluate the disagreement in plaque component detection between manual and automated segmentation, despite the high interscan reproducibility for both methods, we performed a post-hoc analysis in all MRI scans with a mismatch between both methods. All scans in which a large plaque component (MWA > 1 mm^2^) was detected in manual analysis (in both the scan and rescan), but not in automated analysis, as well as all large plaque components detected by automated analysis, but not in manual analysis, were reanalysed by an independent expert (3 years of experience in carotid MRI analysis) for the presence and location of plaque components (Table 2). This reader was blinded for the results from the initial manual and automated analysis.

**Table 2:** Post-hoc manual analysis of patients with a mismatch in the detection of LRNC and calcifications by manual and automated analysis.

	Large components reproducibly present in manual analysis			Large components reproducibly present in automated analysis		
	Total	Missed by automated analysis	Present in post-hoc manual analysis	Total	Missed by manual analysis	Present in post-hoc manual analysis
LRNC	4	0	n/a	10	6	1/6
Calc	14	4	3/4	12	3	2/3

LRNC = lipid-rich necrotic core, Calc = calcification

All four LRNC reproducibly found in manual analysis, were also detected in automated analysis. In contrast, 6 out of 10 large, reproducibly detected LRNC in automated analysis were missed in manual analysis. From those 6, only one LRNC was found in the post-hoc manual analysis. In 14 patients large calcifications in the scan and rescan were found in manual analysis, of which 7 were not detected in automated analysis. Of those 4 calcifications not detected by automated analysis, 3 were again detected in the post-hoc manual analysis. Review of these cases revealed that in 2 cases this mismatch could be explained by the presence of a calcification with an area of >1mm2 in the automated analysis at the location of a large calcification in manual analysis.

## Discussion

In the present study we show a substantial disagreement in the detection of LRNC and/or calcification containing plaques using manual and automated plaque component segmentation. When only high quality scans were included in the analysis, the agreement between both manual and automated analyses raised to acceptable levels. Despite the disagreement between both methods, the interscan reproducibility of both methods was high. Interscan reproducibility decreased for manual analysis, but not for automated analysis, in lower quality scans. These data suggest that the performance of plaque component analysis, both manual and automated, is dependent on scan quality, thereby questioning the clinical applicability of this technique in its current form.

With automated segmentation of plaque components, a LRNC was detected in the scan and/or rescan in 23 of 24 plaques, compared to 6 of 24 for the manual segmentation. It is in line with literature [14,21] that automated plaque segmentation results in the detection of a larger number of small LRNC (MWA <1 mm^2^). In the present study, however, this only explains the disagreement in part, since the agreement between manual and automated segmentation of LRNC remained poor after exclusion of LRNC with a mean wall area of < 1 mm^2^. All of the 4 large, manually detected LRNC (mean MWA 7.4 +/- 3.4 mm2) were also detected in automated analysis. On the other hand, from the 6 large LRNC identified by automated but missed by manual analysis, five were also not detected in the blinded post-hoc manual analysis. These results are indicative of a high sensitivity, but low specificity of automated segmentation of LRNC in the present dataset. Using the MEPPS algorithm, plaque component classification is performed for each voxel based on the relative signal intensity on different MR weightings, the local plaque thickness and the distance to lumen. Noise due to low SNR or motion artefacts in a thickened wall area can thereby potentially be misinterpreted as LRNC by this algorithm. The high reproducibility for automated segmentation in the lower quality scans may thus be a result of reproducible false-positive detection of LRNC. The observed higher agreement in the detection of a LRNC between manual and automated analysis in the higher quality scans (*k* of 0.55 compared to 0.29 in lower quality scans) is in line with the hypothesis of false-positive LRNC detection by automated analysis in lower quality scans. Previous studies using the MEPPS algorithm to study the agreement between manual and automated segmentation of LRNC showed better concordance, with *kappa* values ranging from 0.62 to 0.77 [14,19]. Automated plaque component segmentation software developed by another research group showed variable agreement with manual segmentation. A substantial agreement for LRNC detection compared to manual analysis (*k* = 0.65) found in the initial study[22] could not be reproduced in a second study, in which a lower agreement was found (Spearman r of 0.35) [23].

Also for the detection of calcified plaques, we found a poor agreement between manual and automated segmentation in the present study. This disagreement also remained after excluding calcifications with a plaque area of < 1mm2: More large calcified plaques were detected using manual segmentation (19/24) compared to automated analysis (12/24). These findings are in contrast to three previously discussed studies, were a high agreement (*k =* 0.74[14,19]; r = 0.65[23]) was found between both methods. However, van ‘t Klooster et al[22], also found an absence of agreement between manual and automated detection of calcifications (r = 0.1) using a different automated plaque segmentation algorithm.

Combined, a better agreement between manual and automated plaque component segmentation was expected, since 1) a higher agreement was found in previous studies comparing both methods; 2) both manual [15,21] and automated plaque component segmentation has been validated against the gold standard, histological assessment of plaque composition; and 3) the high interscan reproducibility of both the manual and automated analysis found in the present study, and the high intra- and interobserver reproducibility for both methods reported previously [17,21]. There are several potential explanations for the observed disconcordance between manual and automated segmentation of LRNC and calcification in the present study. First, a contrast-enhanced T1W (CE-T1W) sequence was not implemented in our multi-contrast MRI protocol. Since addition of a CE-T1W sequence has been shown to increase accuracy and reproducibility of LRNC detection and quantification[15,24,25], this may have reduced the performance of both methods in the present study. Second, in contrary to several previous studies[5,15,22] we did not select our scans based on image quality, and therefore differences in scan quality between the present and previous studies can explain the low agreement in part. As we show that both reproducibility of manual segmentation as well as the agreement between manual and automated segmentation are reduced in lower quality scans, the performance of plaque component identification is likely to depend on scan quality. Finally, in all previously discussed studies investigating the accuracy of automated plaque component segmentation software and the agreement with manual segmentation[14,19,22,23], the MRI datasets used for training and validation of the automated segmentation program were derived from the same study cohort or same study center. We are, to the best of our knowledge, the first to apply a validated algorithm to an external dataset, and compare it to manual analysis performed by external experts. This may result in a decline in performance of the measurement by subtle differences in scan quality, MRI acquisition parameters and protocol used for identification of different plaque components.

### Limitations

The present study has several limitations. First, intraplaque haemorrhage was detected in neither automated nor manual analysis, whereby the agreement between both methods for IPH identification could not be tested. The absence of IPH is not surprising, taking into account the low expected prevalence of IPH in our study population mainly consisting of patients with asymptomatic carotid artery stenosis[6]. As IPH is the plaque component with the strongest predictive value for stroke in prospective studies[10], similar studies in populations with a higher prevalence of IPH (i.e. patients with recent stroke) are needed to evaluate the reproducibility and accuracy of manual and automated detection of IPH. Of interest is the agreement between manual and automated analysis in not detecting any IPH. A second important limitation of the present study comprises the lack of a gold standard, preferably histological assessment of plaque components. Therefore, we cannot definitely attribute the lack of agreement between manual and automated segmentation to one of either methods. However, based on our results, we can question the performance of both methods, especially in lower quality scans, as we suggest that automated segmentation results in over classification of plaque regions as LRNC, and the reproducibility of manual plaque component segmentation dramatically decreases in lower quality scans.

### Implications for clinical and research applications of plaque composition analysis

Based on these findings, we can speculate that the the performance of both manual and automated plaque component segmentation critically depends on the quality of the imaging data, though no final conclusion can be drawn in the absence of a gold standard (i.e. histology) [26]. Even in high quality scans, the agreement between manual and automated analysis remains moderate. Combined with the relatively low number of high quality scans in our study, reflecting the difficulties in obtaining high quality carotid MRI scans using the current techniques, this stresses the need for improved imaging and analysis methods. Quantitative imaging techniques applied in the carotid artery [27,28] could potentially overcome these problems and thereby improve accuracy and reproducibility, which is warranted for wider implementation of carotid plaque component analysis for risk stratification in patients with carotid artery stenosis.

## Supporting Information

S1 Table MRI scan acquisition parameters. (DOCX)
