# Supplementary material for: Manual versus Automated Carotid Artery Plaque Component Segmentation in High and Lower Quality 3.0 Tesla MRI Scans
Source: PLoS One. 2016 Dec 8;11(12):e0164267. doi: 10.1371/journal.pone.0164267 (PMC5145140; doi:10.1371/journal.pone.0164267)
Supplement: S1 Table — (DOCX) [file pone.0164267.s001.docx]

**S1 Table. MRI scan acquisition parameters**

| **Parameter** | **Black blood**  **T1w** | **Black blood**  **T2w** | **Black blood**  **PDw** | **Bright blood**  **TOF** |
| --- | --- | --- | --- | --- |
| **Sequence** | TSE | TSE | TSE | FFE |
| **ECG Gating** | End diastole | End diastole | End diastole | Gate delay 200 ms |
| **TR (ms)** | 1 heart beat | 2 heart beats | 2 heart beats | 35 |
| **TE (ms)** | 8 | 50 | 8 | 7 |
| **Resolution (mm)** | 0.25 x 0.25 | 0.25 x 0.25 | 0.25 x 0.25 | 0.25 x 0.25 |
| **Slice thickness (mm)** | 2 | 2 | 2 | 2 |
| **Flip Angle** | 90 | 90 | 90 | 20 |
| **Number of slices** | 8 | 8 | 8 | 8 |
| **Blood suppression** | DIR | DIR | DIR | Inflow suppression |
| **Fat suppression** | SPAIR | SPAIR | SPAIR | None |

Table legend: TOF = time of flight; TSE = turbo spin-echo, FFE = fast field echo, DIR = double inversion-recovery, SPAIR = Spectral Attenuated Inversion Recovery, TR = repetition time, TE = echo time.
